# Functional Constraint Profiling of a Viral Protein Reveals Discordance of Evolutionary Conservation and Functionality

**DOI:** 10.1371/journal.pgen.1005310

**Published:** 2015-07-01

**Authors:** Nicholas C. Wu, C. Anders Olson, Yushen Du, Shuai Le, Kevin Tran, Roland Remenyi, Danyang Gong, Laith Q. Al-Mawsawi, Hangfei Qi, Ting-Ting Wu, Ren Sun

**Affiliations:** 1 Department of Molecular and Medical Pharmacology, David Geffen School of Medicine, University of California, Los Angeles, Los Angeles, California, United States of America,; 2 Molecular Biology Institute, University of California, Los Angeles, Los Angeles, California, United States of America,; 3 Department of Microbiology, Third Military Medical University, Chongqing, 400038, China; 4 AIDS Institute, University of California, Los Angeles, Los Angeles, California, United States of America; University of Arizona, UNITED STATES

## Abstract

Viruses often encode proteins with multiple functions due to their compact genomes. Existing approaches to identify functional residues largely rely on sequence conservation analysis. Inferring functional residues from sequence conservation can produce false positives, in which the conserved residues are functionally silent, or false negatives, where functional residues are not identified since they are species-specific and therefore non-conserved. Furthermore, the tedious process of constructing and analyzing individual mutations limits the number of residues that can be examined in a single study. Here, we developed a systematic approach to identify the functional residues of a viral protein by coupling experimental fitness profiling with protein stability prediction using the influenza virus polymerase PA subunit as the target protein. We identified a significant number of functional residues that were influenza type-specific and were evolutionarily non-conserved among different influenza types. Our results indicate that type-specific functional residues are prevalent and may not otherwise be identified by sequence conservation analysis alone. More importantly, this technique can be adapted to any viral (and potentially non-viral) protein where structural information is available.

## Author Summary

The analysis of sequence conservation is a common approach to identify functional residues within a protein. However, not all functional residues are conserved as natural evolution and species diversification permit continuous innovation of protein functionality through the retention of advantageous mutations. Non-conserved functional residues, which are often species-specific, may not be identified by conventional analysis of sequence conservation despite being biologically important. Here we described a novel approach to identify functional residues within a protein by coupling a high-throughput experimental fitness profiling approach with computational protein modeling. Our methodology is independent of sequence conservation and is applicable to any protein where structural information is available. In this study, we systematically mapped the functional residues on the influenza A PA protein and revealed that non-conserved functional residues are prevalent. Our results not only have significant implication on how functionality evolves during natural evolution, but also highlight the caveats when applying conservation-based approaches to identify functional residues within a protein.

## Introduction

To comprehensively describe the functional roles of a given protein, which are often diverse for many viral proteins and include catalytic activity, intermolecular interactions, and/or cofactor binding, it is necessary to identify the individual functional residues that carry out the biochemical mechanism. Sequence conservation analysis is a common strategy to search for functional residues and is facilitated by the availability of public protein sequence databases [1–3]. The underlying logic is composed of two parts. First, functional residues are essential. Second, essential residues are conserved. However, the reverse may not hold true − conserved residues are not necessary essential. With the extensively studied influenza A virus, several groups have experimentally demonstrated that conserved residues need not be essential for viral replication [4–6]. In addition, a residue shown to be essential for viral replication can also be the result of stability constraints, where the residue is essential for protein stability and expression levels, rather than due to functional constraints [7–10].

Another caveat of sequence conservation analysis is the inefficacy for identifying species-specific functional residues. This issue is often overlooked. During natural evolution, continuous diversification and adaptation leads to the acquisition of new functions. For example, NS1 from influenza B but not influenza A interacts with ISG15 [11]; NS1 from influenza A but not influenza B interacts with CPSF30 [12]. Furthermore, certain phosphorylation sites are not conserved across influenza A and B viruses [13]. In fact, non-conserved functional residues have been demonstrated in various organisms [14–17]. Consequently, when comparing the sequence identities of a set of diverse homologs, as is the case when comparing influenza types A, B, and C, species-specific functional residues may appear as non-conserved residues and be classified as non-functional. As a result, development of a sequence conservation-independent approach is needed to provide an unbiased assessment for the functionality of individual residues and to permit a systematic interrogation of the relationship between functionality and evolutionary conservation.

The influenza A virus PA polymerase subunit consists of a ∼ 25 kD N-terminal domain and a ∼ 55 kD C-terminal domain [18, 19]. Structural information for both domains is available [20–23]. PA forms a heterotrimer complex with two other influenza virus proteins, PB1 and PB2. Together, they function as an RNA-dependent RNA polymerase. The three subunits perform distinct functions, which contribute to the replication and transcription of the viral RNA genome. PB1 binds to the viral promoter and is the catalytic subunit for viral RNA synthesis [24]. PB2 is essential for the transcription of viral RNA and can bind to the 5’ cap of host pre-mRNAs for “cap-snatching” [25–27]. PA is required for both replication and transcription of the viral RNA and contains an endonuclease catalytic site for cleaving the capped RNA primer [28–31]. It has also been reported that PA may be involved in other viral processes, such as viral assembly [32, 33], and may possess protease activity [34, 35]. Recently, several groups have proposed targeting the influenza PA polymerase subunit for antiviral drug development as it is an essential component for viral replication [36–42].

In this study, we have developed a systematic approach that is independent of any prior knowledge in sequence conservation to identify functional residues at single amino acid resolution. In this strategy, we coupled a high-throughput fitness profiling platform with an *in silico* mutant stability prediction. We employed the influenza A virus PA polymerase subunit as the target protein, due to the availability of structural information and the extensive information available for natural sequence variants. The fitness effects of amino acid substitutions were profiled across 94% of all PA protein residues using a novel “small library” approach. Computational modeling predicted the stability effect of all individual substitutions, thus uncovering the structural constraints for individual residues. By integrating the fitness and structural information, we identified known functional sites previously documented in the literature and provide additional insight into the structure-function relationship of the influenza PA protein. We further examined the relationship between evolutionary conservation and functional constraints and show that functional residues are not necessarily conserved. This study not only describes a novel functional annotation platform that provides insight into the relationship between functionality and sequence conservation, but also presents valuable information for drug development and future functional studies of the influenza A virus PA protein. More importantly, this approach has the potential to be adapted for any protein where structural information is available.

## Results

### Design of a high-throughput genetic platform for fitness profiling

High-throughput genetic approaches have been applied to the study of various proteins (reviewed in [43]), which include several from influenza virus and HIV [44–49]. Generally, a mutant library is monitored using deep sequencing, and the relative fitness of each mutation can be inferred by changes in the frequency of mutation occurrence throughout the fitness selection process. Mutant library construction represents a key step in these high-throughput genetic approaches. An ideal mutant library should contain only one point mutation per genome, which poses a challenge for high-throughput mutagenic strategies. Existing approaches have used viral genomes that contain multiple mutations within the mutant library. However, the short read length in current deep sequencing technologies disallows the examination of any possible linkage between distantly placed mutations within each genome. Consequently, genetic interactions between mutations may exist during the selection process, but are not accounted for during the fitness calculation for individual point mutations.

**Fig 1:**
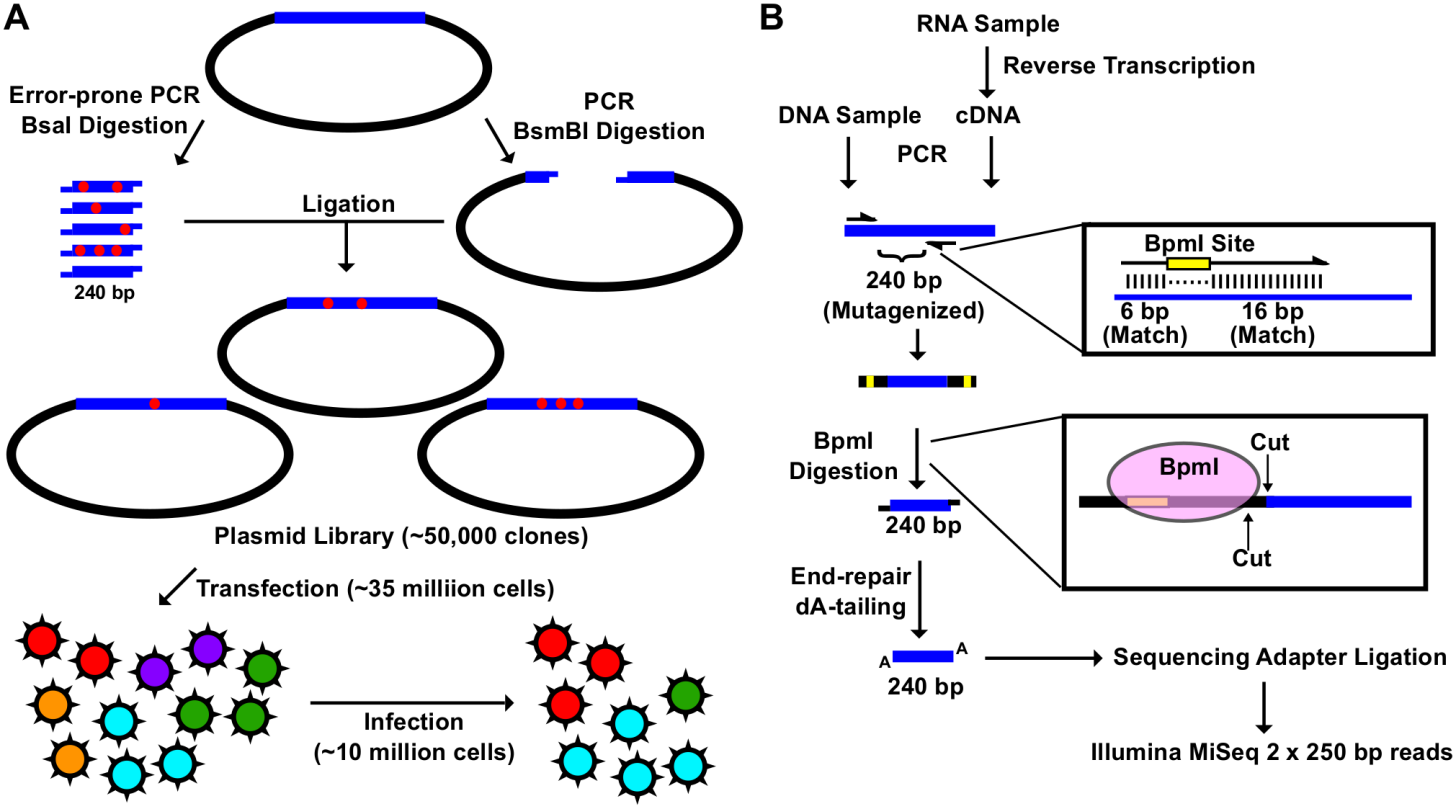
Construction of the mutant libraries. (A) A schematic representation of the fitness profiling experiment is shown. A 240 bp insert was generated by error-prone PCR and BsaI digestion. The corresponding vector was generated by high-fidelity PCR and BsmBI digestion. Each of the nine plasmid libraries in this study consist of ∼ 50,000 clones. Each viral mutant library was rescued by transfecting ∼ 35 million 293T cells. Each infection was performed with ∼ 10 million A549 cells. (B) A schematic representation of the sequencing library preparation is shown. DNA plasmid mutant library or viral cDNA was used for PCR. This PCR amplified the 240 bp randomized region. The amplicon product was then digested with BpmI, end-repaired, dA-tailed, ligated to sequencing adapters, and sequenced using the Illumina MiSeq platform. BpmI digestion removed the primer region in the amplicon PCR, resulting in sequencing reads covering only the barcode for multiplex sequencing and the 240 bp region that was randomized in the mutant library. With this experimental design, the number of mutations carried by individual genomes in the mutant libraries could be precisely determined.

To resolve this drawback in existing high-throughput genetic approaches, we have developed a “small library” strategy (Fig 1A). Each mutant library contains a mutated region that can be covered by a single sequencing read. Here, we generated a 240 bp mutated amplicon by error-prone PCR, which is then cloned into a PCR-generated vector using type IIs restriction enzymes (BsaI or BsmBI). The resulting plasmid mutant library was constructed from ∼ 50,000 clones. A total of nine different “small libraries” for influenza A/WSN/33 PA were constructed. Together, these nine “small libraries” covered the entire PA gene. Each viral mutant library was rescued by transfecting the plasmid mutant library with the other seven wild type (WT) plasmids of the influenza A/WSN/33 eight-plasmid reverse genetic system [50]. A549 cells were then infected with the viral mutant library for 24 hours.

The plasmid mutant libraries (DNA library), post-transfection viral mutant libraries (transfection), and post-infection viral mutant libraries (infection) were subjected to deep sequencing. In this study, we included a technical replicate for sequencing the DNA library, a biological replicate for transfection, and a biological replicate for infection to estimate the reproducibility of individual steps (S1 Fig). In addition, we also sequenced the WT PA plasmid as a control.

The amplicon sequencing library was prepared for the Illumina MiSeq 250 bp paired-end sequencing, using either DNA (DNA library or WT plasmid) or cDNA (transfection or infection) (Fig 1B). For each “small library”, the 240 bp mutated region was amplified by a primer pair that contained a BpmI restriction site. A subsequent BpmI digestion excised the primer region from the PCR amplicon. As a result, the entire 240 bp mutated region would be covered by both forward and reverse reads (S2 Fig). This enabled sequencing error correction by read-pairing. We obtained a coverage of at least 20,000 (range = 20,128 to 965,488) for each sequencing library (S3 Fig).

### Point mutation fitness profiling of influenza PA

The design of our high-throughput genetic platform enables us to examine the mutation in individual genomes. On average, 44% (range = 25% to 76%) of viral genomes contain no mutation (i.e. WT), 33% (range = 20% to 36%) of viral genomes contain a single mutation, and 23% (range = 3% to 42%) of viral genomes contain at least two or more mutations (S4 Fig). While a fraction of the genomes in the mutant library contain more than one mutation due to the nature of error-prone PCR, they were filtered out for downstream analysis. Occurrence frequency for each point mutation was computed from genomes that contained only one mutation. This allowed a precise fitness calculation for individual point mutations without complication by genetic interactions that may exist with additional mutations. Individual point mutations exhibited an occurrence frequency of 0.04% (range = 0% to 0.3%) across all DNA libraries. Whereas the mutation frequency obtained from sequencing the WT plasmid, which served as a control for sequencing error rates, was 0.005% (range = 0% to 0.07%) (S5 Fig).

**Fig 2:**
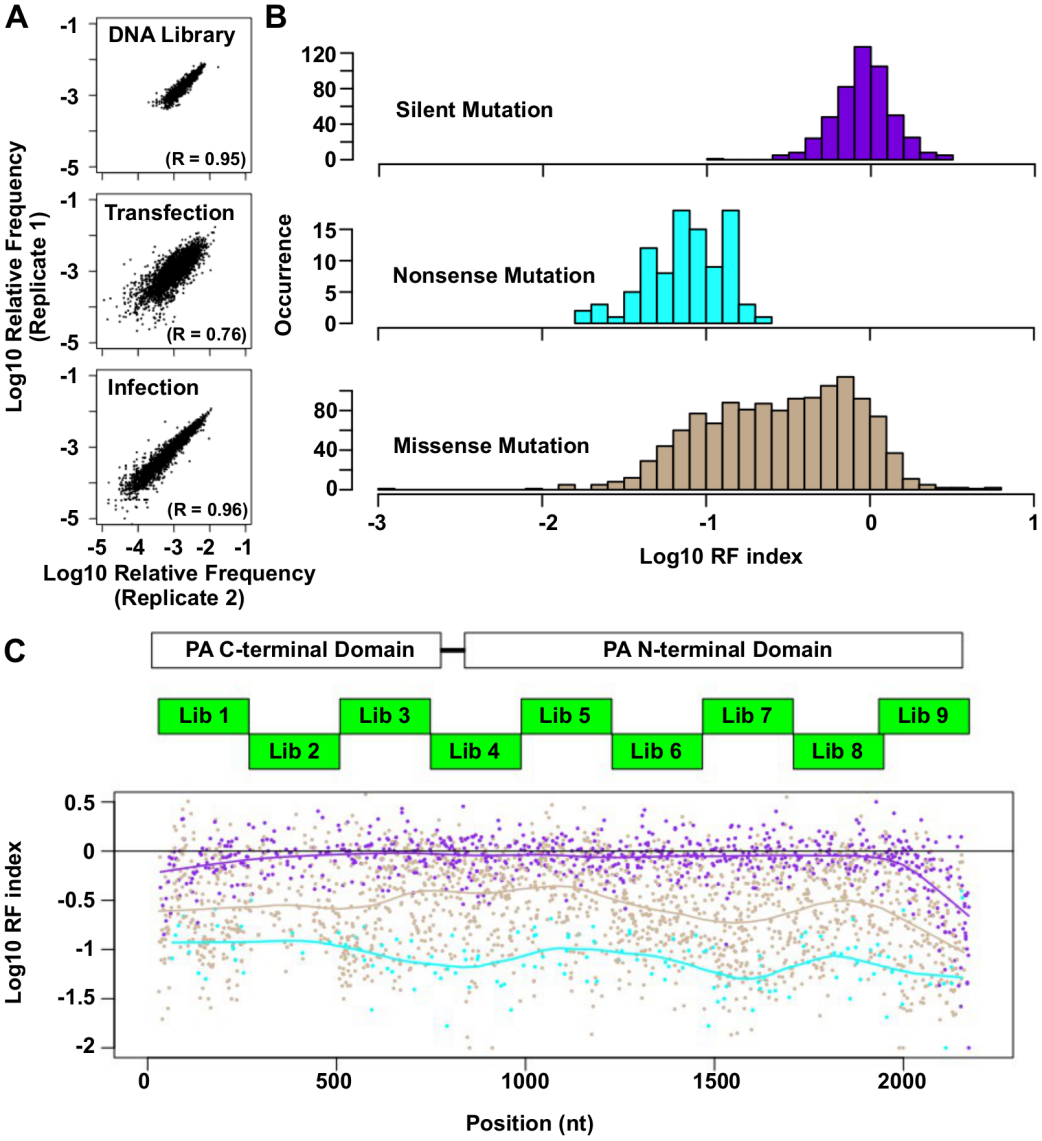
Fitness profiling of PA influenza virus polymerase subunit. (A) Correlations of log_10_ relative frequency of individual point mutations between replicates are shown. Relative frequency_*mutationi*_ = (Occurrence frequency_*mutationi*_)/(Occurrence frequency_*WT*_) (B) Log_10_ RF indices for silent mutations, nonsense mutations, and missense mutations are shown as histograms. Point mutations located at the 5 terminal 400 bp and 3 terminal 400 bp regions are not included in this analysis to avoid complication by the vRNA packaging signal [93, 94]. (C) The locations of the PA C-terminal domain and the PA N-terminal domain are shown as white boxes. The locations of the mutated regions in each mutant library are shown as green boxes. Log_10_ RF indices for individual point mutations are plotted across the PA gene. Each point mutation is colored coded as in panel B. Purple: silent mutations; Cyan: nonsense mutations; Brown: missense mutations. A smooth curve was fitted by loess and plotted for each point mutation type.

Comparison of the relative frequency of individual point mutations between replicates was performed to assess the reproducibility of our “small library” high-throughput genetic platform (see Materials and Methods for the calculation of relative frequency). A Pearson’s correlation of 0.95 was obtained for the technical replicate of DNA library, 0.76 for the biological replicate of transfection, and 0.96 for the biological replicate of infection (Fig 2A). The strong correlations between replicates validated the design of our high-throughput genetic platform. Only those point mutations with an occurrence frequency of ≥ 0.03% in the DNA library were included in the downstream analysis, which covered 42% of all possible point mutations on the PA gene, to avoid fitness calculations being obscured by sequencing errors. The relative fitness index (RF index) was used as a proxy to estimate the fitness effect for each point mutation.

$$\text{RF index }=\;({\text{Relative frequency}}_{infection}{\text{/(Relative frequency}}_{DNAlibrary})$$

The RF index of silent mutations (mean = 0.98) was significantly higher than that of nonsense mutations (mean = 0.09) (P < 2e^−16^, two-tailed Student’s t-test). Furthermore, the RF index distributions of silent mutations versus nonsense mutations were well-separated (Fig 2B), validating that fitness selection was taking place. The fitness effects of substitutions were profiled across 94% of all amino acid residues in PA. The fitness profiling data is shown in Fig 2C.

### Combining high-throughput fitness profiling with mutant protein stability prediction identifies functional sites at single amino acid resolution

Next we aimed to identify amino acid residues that were functionally essential, but not structurally important. Essential residues in viral replication can be systematically mapped by high-throughput fitness profiling experiments [46–48, 51–53]. However, fitness profiling only quantifies essentialness, but does not partition the structural versus functional role of individual residues. Several studies have shown that mutating functional residues imposed minimum stability cost to the proteins in which they reside [54–58], suggesting that functional residues can be pinpointed by identifying substitutions that are deleterious to the virus but not destabilizing to the protein.

**Fig 3:**
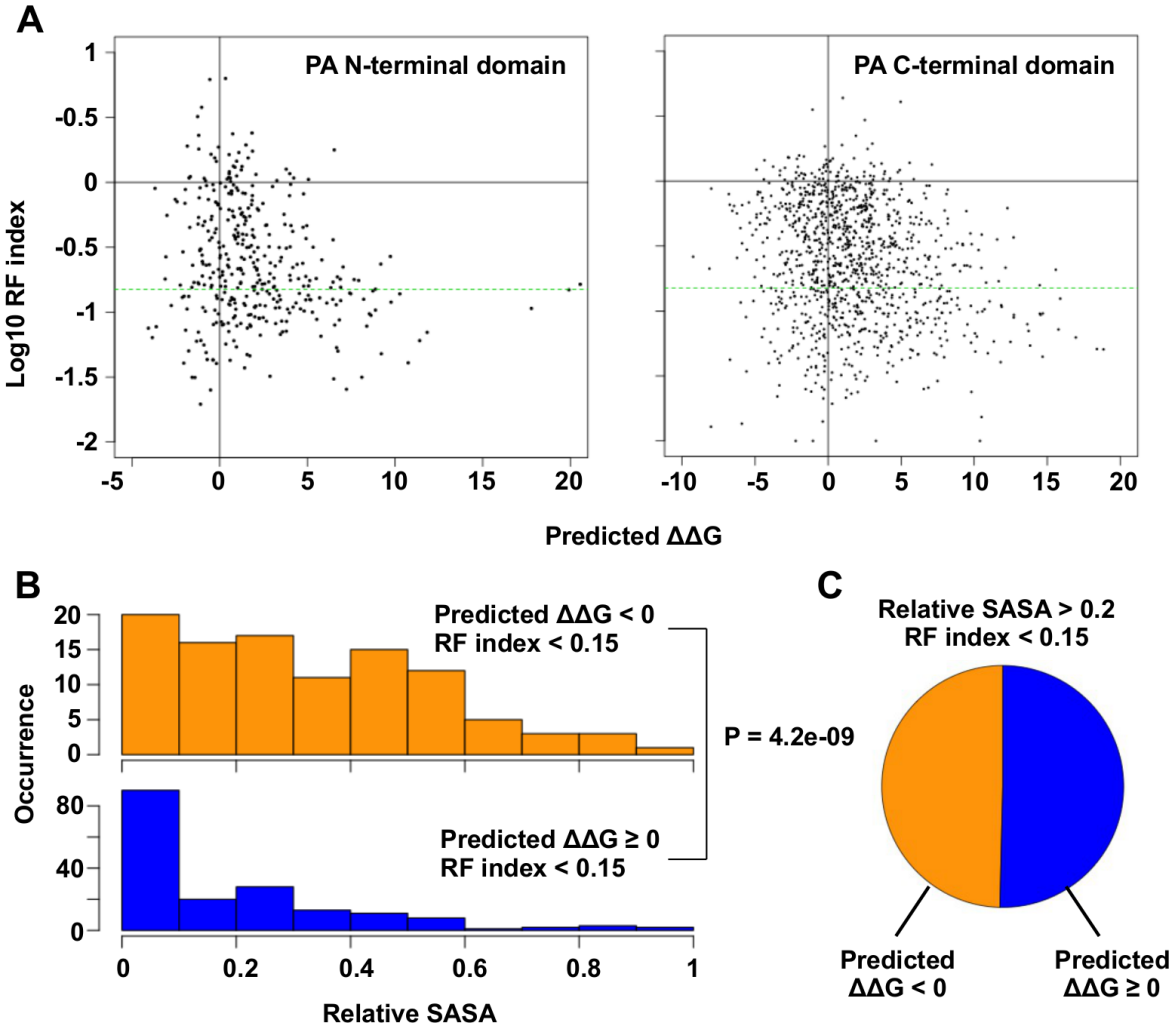
Systematic identification of functional residues. (A) Predicted ΔΔG for each point mutation is plotted against the log_10_ RF index. The horizontal green line represents the RF index cutoff used in this study, RF index = 0.15. For the N-terminal domain, the Spearman’s rank correlation between log_10_ RF index and Predicted ΔΔG is -0.20 (P = 1.3e^−4^). For the C-terminal, the Spearman’s rank correlation between log_10_ RF index and Predicted ΔΔG is -0.18 (P = 6.8e^−10^). (B) The distributions of relative SASA are shown for residues that carried at least one substitutions of interest (RF index < 0.15 and a predicted ΔΔG < 0) and for residues that did not carry any substitutions of interest. (C) This analysis is performed on those solvent exposed residues (relative SASA > 0.2) that carried a deleterious mutation (RF index < 0.15). The pie chart is showing the fraction of residues that carried a substitution of interest (ΔΔG < 0) and those did not (ΔΔG ≥ 0).

Using Rosetta software we predicted the effect of individual substitutions on protein stability. We used the parameters from row 16 of Table I in Kellogg et al., which has been shown to give a correlation of 0.69 with experimental data and a stability-classification accuracy of 0.72 [59, 60]. We were able to identify substitutions that had a low RF index, but did not destabilize the protein (Fig 3A). We hypothesized that these residues had large functional constraints with little structural effects to the protein upon substitution. To identify the substitutions of interest, a cutoff was set at an RF index < 0.15 (based on the separation point of silent mutations and nonsense mutations) and a predicted ΔΔG < 0 (not destabilizing). A total of 32 substitutions (22 unique residues) in the PA N-terminal domain and 110 substitutions (81 unique residues) in the PA C-terminal domain satisfied these criteria.

A number of functional residues in the PA protein have been experimentally characterized in the literature (S1 Table). Out of 32 substitutions of interest in the PA N-terminal domain, eight were at residue positions that carried known biological functions. This included five substitutions in the endonuclease active site (E80V, E80G, E80K, E119V, K134) [20, 21], and six substitutions in the cRNA promoter binding site (E166D, R170W, R170M, R170K, T173I, T173A) [18, 61]. We also found multiple residues with known biological functions among the 110 substitutions of interest in the C-terminal domain. This included a substitution at a residue required for endonuclease activity (H510R) [28], a substitution at a residue required for small viral RNA (svRNA) binding (R566W) [62], four substitutions at residues required for viral genome replication (E410V, E524V, K539M, K539E) [28], and six substitutions at the PB1-binding site (N412I, N412Y, Q670R, Q670L, F710I, F710Y) [22, 23]. For all residues that carry a deleterious substitution (RF index < 0.15), residues identified as functional residues (ΔΔG < 0) had a larger relative SASA (solvent accessible surface area) versus amino acid positions that were not (P = 4.2e^−9^, two-tailed Student’s t-test) (Fig 3B). This indicates that the identified functional residues were mostly surface exposed, as expected if they mediate possible interactions with biomolecules. In fact, ∼ 50% of the solvent exposed residues that carried a deleterious mutation (relative SASA > 0.2 and RF index < 0.15) were identified as functional residues (Fig 3C). Since our mutagenesis technique was based on error-prone PCR, which results in a non-comprehensive sampling of all the possible amino acid substitutions at each site, there may be some functional substitutions that were not sampled in our study. Nonetheless, these results demonstrate the feasibility of combining high-throughput fitness profiling with mutant stability prediction to identify functional sites at single amino acid resolution.

### Identification of residues in the PA C-terminal region with functions unrelated to polymerase activity

**Fig 4:**
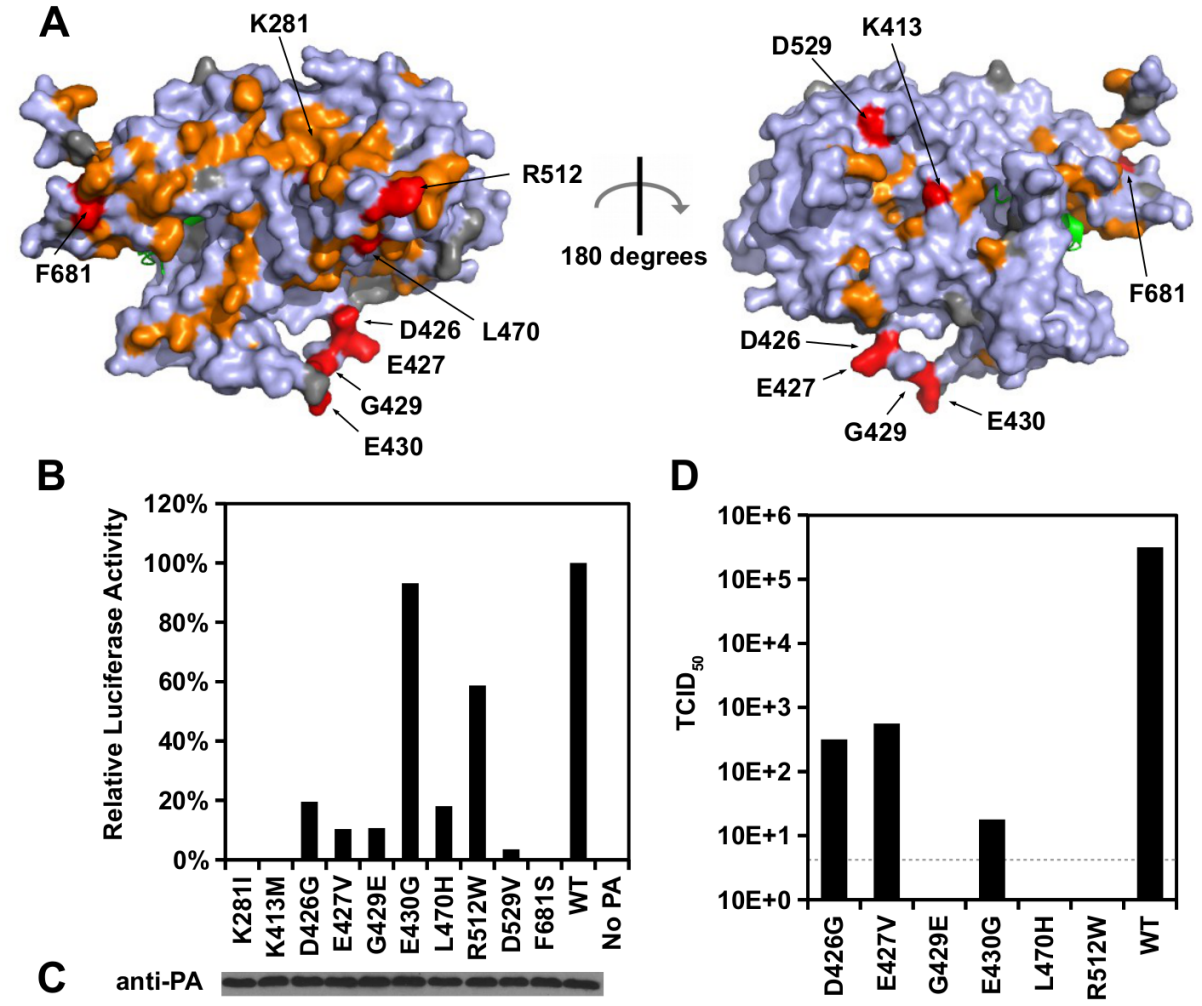
Identification of PA residues that carry non-polymerase functions. (A) Locations of substitution with an RF index < 0.15 and a predicted ΔΔG < 0 are colored in orange or red respectively. Mutations that were individually reconstructed and analyzed in this study are labeled and colored in red. Residues that were not covered in our profiling data are colored in grey. For PB1, only the N-terminal helix is structurally available in this PDB file, and is colored in green. PDB ID: 2ZNL [23]. (B) The effects of different PA point mutations on influenza polymerase activity were measured using an influenza A virus-inducible luciferase reporter assay [63]. Error bar represents the standard deviation of three biological replicates. (C) The expression level of each PA mutant was tested by immunoblot analysis. (D) TCID_50_ of the rescued mutant or WT viruses was measured.

Since the PA C-terminal region’s structure-function relationship remains largely unclear, we aimed to identify functional residues in this region to provide additional insight into the role of PA during viral replication. Ten previously uncharacterized substitutions with an RF index < 0.15 and a predicted ΔΔG < 0 were individually reconstructed and analyzed. Their spatial locations were distributed throughout the PA C-terminal domain (Fig 4A and S6 Fig). The effect of these substitutions on the influenza polymerase activity was tested using an influenza A virus-inducible luciferase reporter assay [63] (Fig 4B). Three substitutions, K281I, K413M, and F681S, completely abolished the influenza polymerase activity. This defect is unlikely to be a protein destabilizing effect since all ten mutants analyzed did not alter protein expression levels as compared to WT (Fig 4C). The fact that nine out of ten mutants had a decrease in polymerase activity as compared to WT further validated our high-throughput approach in identifying deleterious mutations.

Interestingly, we found six substitutions (D426G, E427V, G429E, E430G, L470H, and R512W) that retained > 10% of the WT influenza polymerase activity (Fig 4B). A rescue experiment was performed using the influenza A/WSN/33 eight-plasmid reverse genetic system [50]. Unexpectedly, R512W, which had ∼ 60% of the WT polymerase activity, completely abolished the production of viral particles (Fig 4D). In addition, E430G, which had a polymerase activity comparable to WT, displayed a four-log drop in virus titer as compared to WT. In contrast, although D426G and E427V displayed a polymerase activity that was only ∼ 10%-20% of WT, each could produce a much higher amount of infectious virus as compared to other substitutions in this set (one-log to two-log higher titers as compared to E430G). Our results suggest that the E430G and R512W substitutions each had a functional defect that is unrelated to the polymerase activity.

E430G and R512W were selected for further functional characterization because they exhibited the strongest polymerase activity among all the individually analyzed substitutions, despite their defect in producing infectious virus. During a viral rescue experiment, there was an accumulation of viral copy number in the supernatant for WT, but not for the E430G and R512W viral mutants (S7A Fig). In contrast, both mutants displayed an accumulation of intracellular viral copy number similar to WT (S7B Fig). At 72 hours post-transfection, the HA titer of R512W and E430G was undetected, indicating viral particles were present at a very low amount, if present (S7C Fig). These results further confirm that E430G and R512W have a defect that is unrelated to polymerase activity.

### Structural analysis of the single residue functional profile

When this study was initiated, PA was the only influenza polymerase subunit with structural information available. The structural information for the other two influenza polymerase subunits, PB1 and PB2, were largely unknown. Nonetheless, after the completion of this study, the crystal structure of the complete influenza A virus polymerase complex bound to the viral RNA promoter has been published [64], which provides an independent reference to validate and interpret our data.

**Fig 5:**
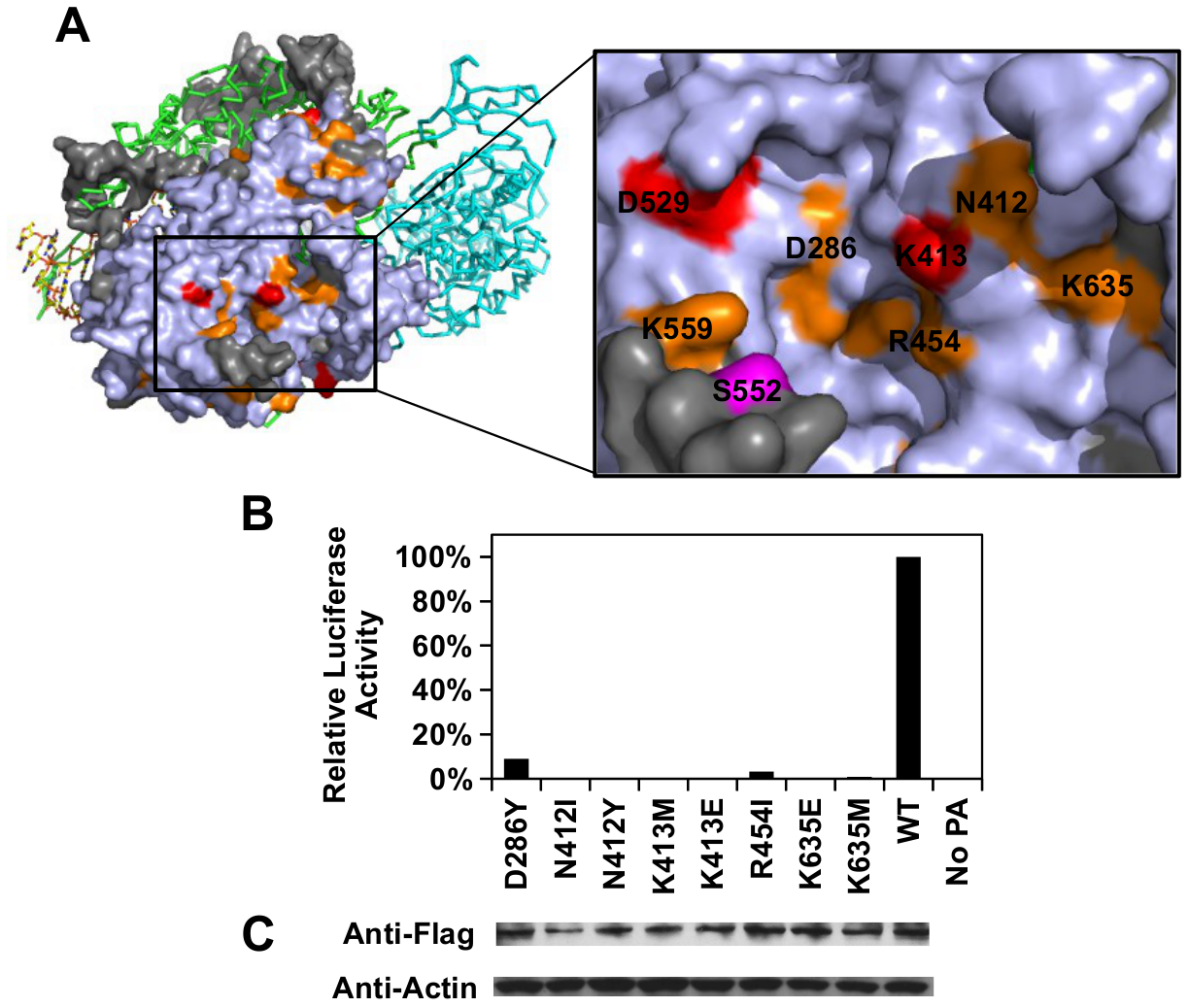
Structural analysis of putative functional residues. (A) The location of a putative functional subdomain is shown on the structure of the influenza polymerase heterotrimeric complex (PDB: 4WSB) [64]. For PA, residues were colored as according to the scheme presented in Fig 4. A putative host determinant residue, S552, is colored in magenta. Note, residue 559 carries an arginine [R] instead of a lysine [K] on the PA of A/WSN/33. (B) The effects of different PA point mutations on influenza polymerase activity were measured using an influenza A virus-inducible luciferase reporter assay [63]. Error bar represents the standard deviation of three biological replicates. (C) The expression level of each C-terminal Flag-tagged PA mutant or WT was tested by immunoblot analysis. The expression level of actin was served as a loading control.

Our functional profile identified a subset of PA residues that interact with PB1 (S8A Fig), PB2 (S8B Fig), and the viral RNA promoter (S9 Fig). Moreover, six out of the 10 validated functional residues participate in these interaction interfaces: − D426, E427, and F681 interacted with PB1; L470 interacted with PB2; K281 and R512 interacted with the viral RNA promoter. Our data also identified functional residues that were not involved in polymerase complex formation or RNA binding activity. For example, E430 did not interact with either PB1, PB2, or the viral RNA promoter (S10 Fig). This is consistent with our data that E430 is involved in a non-polymerase function. In addition, a putative functional subdomain independent of the polymerase-interacting surface was identified in our functional profiling data. This putative functional subdomain is composed of a series of charged or polar residues − D286, N412, K413, R454, D529, K559, and K635. Interestingly, this patch of functional residues was adjacent to residue 552, which has been shown to be a host-specific determinant [65]. This indicates a possible biological significance of the putative functional subdomain we identified. Consistently, substitutions at positions D286, N412, K413, R454, D529, and K635 were shown to abolish the polymerase activity in our validation experiment (Fig 5B-5C), further confirming the functional importance of this subdomain in viral replication.

Overall, our profiling data is consistent with the polymerase complex-viral RNA promoter complex structural data, which provides an independent validation of our approach.

### Relationship between functional constraints, structural constraints, and evolutionary conservation

There are three types of influenza viruses, namely type A, B, and C. Phylogenetic analysis indicates that PA displays a high inter-type diversity (evolutionary distance among viral strains within the same influenza type), while the intra-type diversity is limited (evolutionary distance between viral strains of different influenza types) (S11 Fig). The average inter-type amino-acid sequence identity is < 40% and that of intra-type is > 95%. The huge divergence among different types of influenza viruses leads us to hypothesize that a significant number of functional residues are type-specific and are non-conserved across different influenza types. Consequently, we aimed to interrogate the relationship between functional constraints, structural constraints and evolutionary conservation. In this study, sequence conservation for each residue was computed using Shannon’s entropy [66]. The higher the entropy, the less conserved a residue is. Here, we divided all profiled residues into three groups: 1) Functional residues, which had at least one substitution that displayed an RF index < 0.15 and a predicted ΔΔG < 0.2) Structural residues, which did not satisfy the condition of functional residues but had at least one substitution that displayed an RF index < 0.15. 3) “Other” residues, which contained all other profiled residues that were neither functional nor structural residues (i.e. all profiled substitutions at “other” residues displayed an RF index ≥ 0.15).

**Fig 6:**
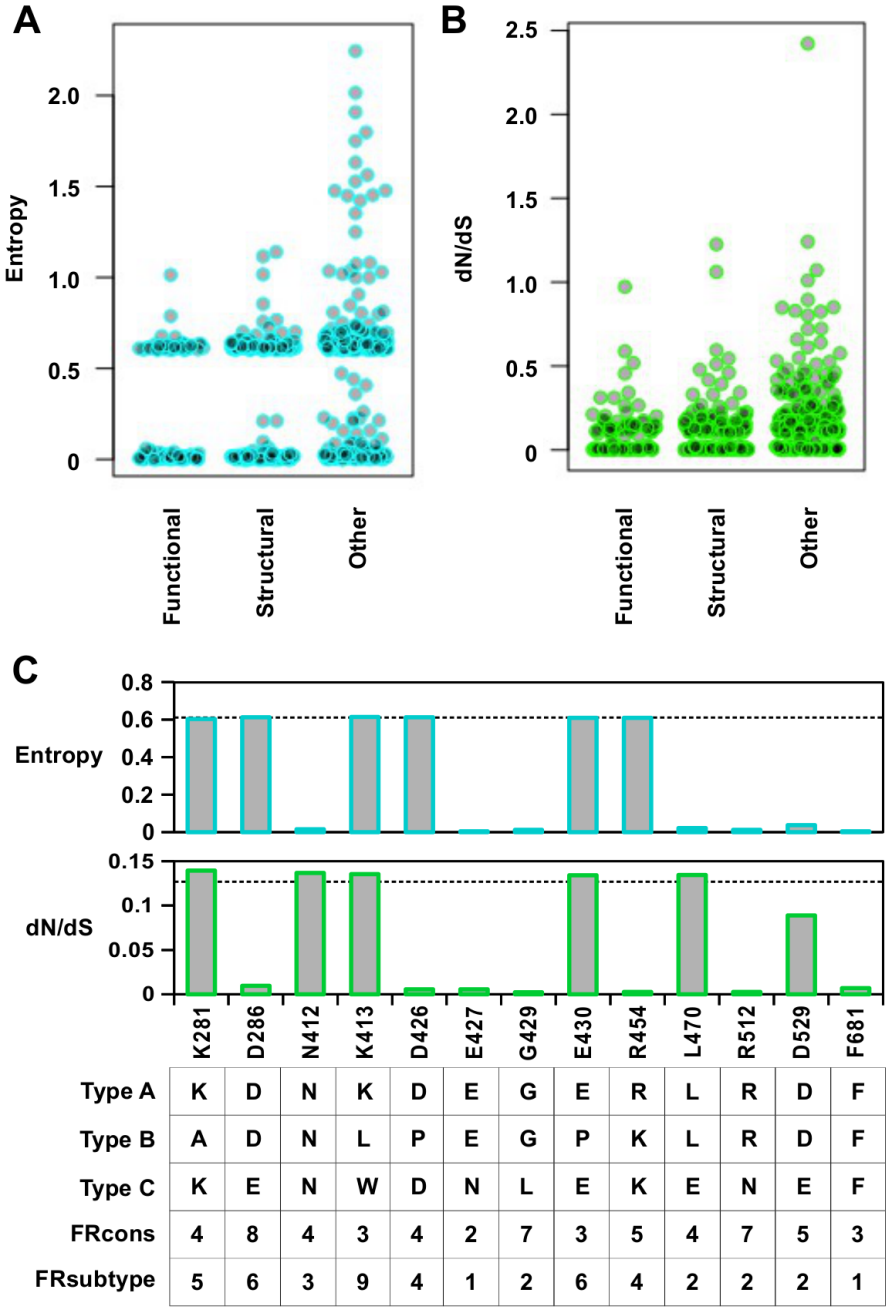
Sequence entropy analysis. (A) Distribution of sequence entropy for functional residues, structural residues, and “other” residues. (B) Distribution of dN/dS for functional residues, structural residues, and “other” residues. (C) Sequence entropy, dN/dS, the natural concensus residue, FRcons category, and FRsubtype category are shown for the validated functional residues in this study. The dashed line indicated the median value across the entire PA segment. For FRcons and FRsubtype, we considered a residue with a category of ≥ 8 as a hit (a total of 72 residues were identified as a hit in each of these two methods).

The entropy calculation was performed on a multiple sequence alignment of 3837 strains from different influenza types (Fig 6A). In general, functional residues were more conserved than structural residues (P = 0.032, Wilcoxon rank-sum test), and structural residues were more conserved than “other” residues (P = 2.9e^−9^, Wilcoxon rank-sum test) (S12 Fig). From this analysis, 58% of functional residues, 43% of structural residues, and 26% of “other” residues were highly conserved (entropy < 0.1). This indicates that a significant number of functional residues are not conserved across the different types of influenza virus.

We further computed a phylogenetic-based dN/dS analysis on each codon across the influenza A virus PA coding sequence with FUBAR [67] (Fig 6B). A mild, yet statistically significant, correlation was detected between dN/dS and RF index (Spearman’s rank correlation = 0.38, P < 2.2e^−16^) (S13A Fig). On average, functional residues and structural residues had a lower dN/dS as compared to “other” residues (P = 7.2e^−8^ and P = 1.5e^−8^ respectively) (S13B Fig). However, the difference of dN/dS between functional residues and structural residues was not significant (P = 0.57). This result shows that dN/dS may not be a good indicator to distinguish functional residues from structural residues. In addition, some functional residues exhibited a dN/dS that was well within the range of “other” residues, demonstrating that some functional residues could not be identified by dN/dS analysis alone. The utility of dN/dS is largely determined by the phylogenetic depth of the sequences being analyzed. In fact, it has been shown that when the genetic diversity is low, as is the case of PA protein sequences from type A influenza virus, dN/dS becomes less sensitive to purifying selection [68], and may not be able to identify functional residues.

We next examined individual residues validated in this study. Among the 13 validated functional residues, three (K281, K413, and E430) had both entropy and dN/dS at the median level (Fig 6C). Moreover, these residues are not conserved across different influenza types. These results confirm that functional residues may not be identified by phylogenetic-based analysis alone. As expected, sequence conservation-based functional site prediction software was unable to predict these functional residues. We tested three software approaches, firestar [69] and two classification schemes under FRpred [70, 71], namely FRcons and FRsubtype. FRcons and FRsubtype were each able to identify only one of our validated functional residues (D286 for FRcons and K413 for FRsubtype, respectively) using a category cutoff of ≥ 8. Firestar was not able to identify any of our validated functional residues. Furthermore, out of a set of 28 functional residues identified in the literature (S1 Table), our approach identified 12, whereas FRcons, FRsubtype, and firestar were only capable of identifying 4, 2 and 5 functional residues, respectively. This comparison demonstrates that our methodology can outperform phylogenetic approaches in identifying functional residues.

### Structural basis of type-specific functional residues

**Fig 7:**
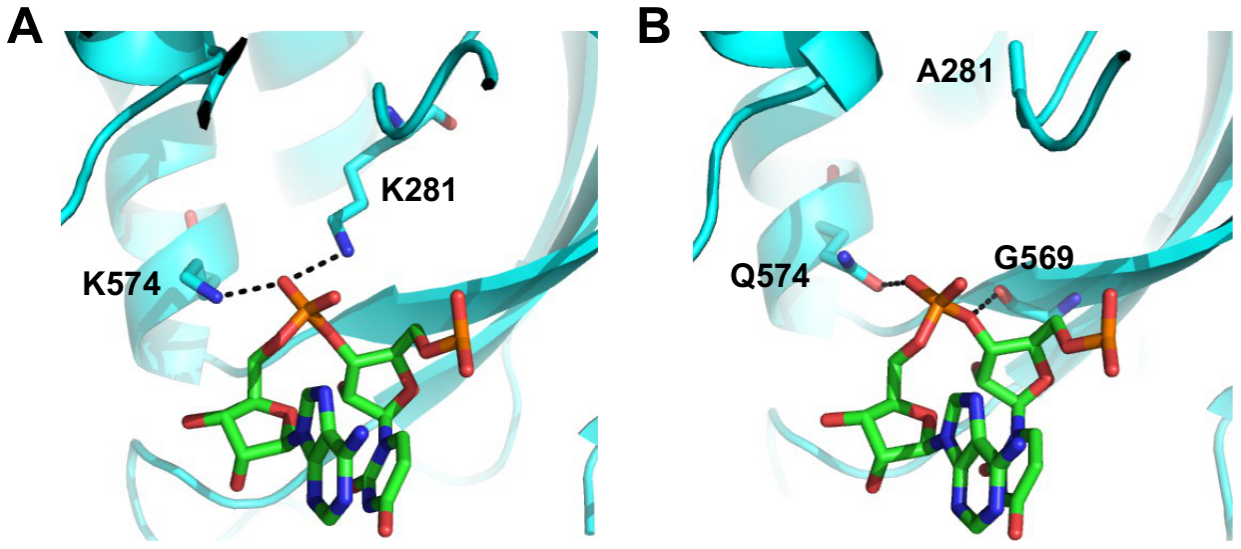
Structure-function relationship of residue 281. (A) The interaction of influenza A PA with the RNA phosphate backbone located between base 3 and 4 is shown. RNA is colored in green. PA is colored in cyan. Hydrogen bonds are represented by dotted black lines. Numbering of residue position is based on A/WSN/33. Conversion of residue position numbering is described in S3 Table. (B) The interaction of influenza B PA with the RNA phosphate backbone located between base 3 and 4 is shown. RNA is colored in green. PA is colored in cyan. Hydrogen bonds are represented by dotted black lines. Numbering of residue position is based on A/WSN/33. Conversion of residue position numbering is described in S3 Table.

We aimed to further investigate the structural basis of type-specific functional residues. The RNA binding function is required for viral replication and is conserved among type A and B influenza viruses. In the validation above, substituting lysine [K] to isoleucine [I] at residue 281 completely abolished the polymerase activity. This highlights the importance of the hydrogen bond formed between K281 and the RNA phosphate backbone in the influenza A virus (Fig 7A and boxed in S14 Fig). However, PA K281 is not conserved between type A and B influenza viruses. All influenza B viruses carry an alanine [A] at residue 281, which is unable to form a hydrogen bond with the RNA backbone. The critical hydrogen bond mediated by K281 in influenza A virus is replaced by the main chain of G569 in the influenza B virus (Fig 7B and boxed in S15 Fig). In fact, structural analysis indicates that type A [64] and B [72] influenza viruses display different hydrogen bonding patterns between PA and the viral RNA promoter (S14 Fig and S15 Fig). Thus, conserved functions may not necessarily require conserved functional residues.

Together, these analyses show that while certain functional residues were completely conserved among different types of influenza viruses, a significant number of residues that mediate critical viral functions may not be conserved, and suggests that some residues may have acquired functionality in recent evolutionary history.

## Discussion

Traditionally, sequence conservation is the common approach for identifying functional residues. In this study, we coupled two high-throughput techniques, experimental fitness profiling and *in silico* mutant stability prediction, to systematically identify functional residues in the influenza A virus PA protein. This strategy provided a direct measure of essentialness and enabled the partitioning of functional constraints versus structural constraints at each residue position. This approach is independent of any prior knowledge of sequence conservation. Therefore, it is devoid of the caveats associated with sequence conservation analysis and possesses the power to identify species-specific functional residues. A number of functional residues identified in this study, are not completely conserved across different types of influenza viruses, suggesting that even functional residues may not be conserved. This disparity between conservation and function highlights the power of our approach to identify functional residues that may not be identified by traditional sequence conservation analysis alone.

We anticipate that this method can be further improved as the accuracy of mutant stability prediction methodology improves. It has been shown that although most force fields exhibit a correct trend in ΔΔG prediction, the precision is still lacking as compared to experimental methods [73]. For example, in this study, N412I decreases protein expression levels, despite being predicted as a stabilizing mutant. In addition, it is known that most proteins are able to buffer a small destabilizing effect without becoming unfolded, and hence without attenuating the fitness [74, 75]. As a result, understanding the stability buffer margin will help to determine the optimal ΔΔG cutoff in our approach. It is also known that many proteins have multiple conformations, which may further complicate the ΔΔG prediction. Together, these caveats may explain the weak correlation between the predicted ΔΔG and RF index in this study. To obtain a more accurate measurement of protein stability, high-throughput experimental analysis on protein stability may provide an alternative [76, 77]. All the advances stated above will improve the accuracy of our platform in identifying functional residues within a target protein.

During natural evolution, continuous accumulation of protein mutations drives speciation and divergence from the common ancestor. The genomic plasticity of an evolving species permits the acquisition of new function through mutations [78]. Evolution of a new function has been demonstrated in bacteriophage *λ* within an experimental timescale [79], and a long-term evolution experiment on *Escherichia coli*[80]. Therefore, it is not surprising to see species-specific function even in recently separated species. Based on the sequence comparison of hemagglutinin, it was estimated that type A and B influenza virus diverged from type C ∼ 8,000 years ago, whereas type A influenza virus diverged from type B ∼ 4,000 years ago [81]. This length of time is sufficient for the influenza virus to develop a type-specific function as exemplified by type-specific virus-host interactions in NS1 [11, 12]. Furthermore, conservation of protein function does not necessarily support that sequence conservation exists at the primary sequence level, which is evidenced by the differences between the nuclear localization signal of influenza A and B NP proteins [82, 83]. In fact, this study reveals that type-specific functional residues are prevalent in the influenza virus PA protein. These results not only provide insight into how functional residues evolve through species diversification, but also highlight the caveats encountered when identifying functional sites from conservation-based approaches.

In the past decade, proteins from different medically important viruses, such as influenza, HIV, and HCV, have been crystallized [84–86]. The approach described in this study systematically integrates the available structural information with mutation fitness information to examine the structure-function relationship of a viral protein of interest and to map functional subdomains. Profiling datasets will facilitate functional characterization of the protein of interest, and will promote targeted drug discovery and rational drug design. The emergence of drug resistant mutations is a major challenge for antiviral drug development. Therefore, it is important to target functional subdomains that are less tolerable to substitution to increase the genetic barrier for developing drug resistant mutations. Our profiling technique can help locate such functional subdomains that are suitable for drug development. More importantly, our technique can potentially be adapted to study any protein, provided the relevant structural information is available.

## Materials and Methods

### Construction of mutant libraries and individual point mutations

The PA plasmid mutant libraries were created by performing error-prone PCR on the PA segment of the eight-plasmid reverse genetics system of influenza A/WSN/1933 (H1N1) [50]. To generate the mutated insert, we PCR-amplified regions of the PA gene from pHW2000-PA plasmid with error-prone polymerase Mutazyme II (Stratagene, La Jolla, CA) according to the manufacturer’s instructions. The following primers were used:

Library 1 insert: 5’-CAG GTC TCA TCA AAA TGG AAG ATT TTG TGC GA-3’ and 5’-CAG GTC TCA ATA CTG TTT ATT ACT GTC CAG GC-3’
Library 2 insert: 5’-CAG GTC TCA TCG AGG GAA GAG ATC GCA CAA TA-3’ and 5’-CAG GTC TCA CTG GTT TTG ATC CTA GCC CTG CT-3’
Library 3 insert: 5’-CAG GTC TCA CCG ACT ACA CTC TCG ATG AAG AA-3’ and 5’-CAG GTC TCA TTT ACT TCT TTG GAC ATT TGA GA-3’
Library 4 insert: 5’-CAG GTC TCA ACG GCT ACA TTG AGG GCA AGC TT-3’ and 5’-CAG GTC TCA TAA TTT GGA TTT ATT CCC TTT TC-3’
Library 5 insert: 5’-CAG GTC TCA AAC CCA ATG TTG TTA AAC CAC AC-3’ and 5’-CAG GTC TCA GCC TTG TTG AAC TCA TTC TGA AT-3’
Library 6 insert: 5’-CAG GTC TCA AAT TGA GGT CGC TTG CAA GTT GG-3’ and 5’-CAG GTC TCA CCC TCC TTA GTT CTA CAC TTG CT-3’
Library 7 insert: 5’-CAG GTC TCA ATT TCC AAT TAA TTC CAA TGA TA-3’ and 5’-CAG GTC TCA TTA ATT TTT GAG GTT CCA TTT GT-3’
Library 8 insert: 5’-CAG GTC TCA GGC CTA TGT TCT TGT ATG TGA GG-3’ and 5’-CAG GTC TCA TGT GGA GAT GCA TAC AAG CTG TT-3’
Library 9 insert: 5’-CAG GTC TCA GAA GGT CTG CAG AAC TTT ATT GG-3’ and 5’-CAG GTC TCA GGA CAG TAT GGA TAG CAA ATA GT-3’

The corresponding vector for each of the nine mutant library was generated by PCR using the following primers:

Library 1 vector: 5’-CAC GTC TCT TTG AAT CAG TAC CTG CTT TCG CT-3’ and 5’-CAC GTC TCA GTA TTT GCA ACA CTA CAG GGG CT-3’
Library 2 vector: 5’-CAC GTC TCC TCG ATT ATT TCA AAT CTG TGC TT-3’ and 5’-CAC GTC TCA CCA GGC TAT TCA CCA TAA GAC AA-3’
Library 3 vector: 5’-CAC GTC TCG TCG GCC TTT GTG GCC ATT TCC TC-3’ and 5’-CAC GTC TCG TAA ATG CTA GAA TTG AAC CTT TT-3’
Library 4 vector: 5’-CAC GTC TCG CCG TTC GGT TCG AAT CCA TCC AC-3’ and 5’-CAC GTC TCA ATT ATC TTC TGT CAT GGA AGC AA-3’
Library 5 vector: 5’-CAC GTC TCG GGT TCC TTC CAT CCA AAG AAT GT-3’ and 5’-CAC GTC TCA AGG CAT GTG AAC TGA CCG ATT CA-3’
Library 6 vector: 5’-CAC GTC TCC AAT TCT GGT TCA TCA CTA TCA TA-3’ and 5’-CAC GTC TCG AGG GAA GGC GAA AGA CCA ATT TG-3’
Library 7 vector: 5’-CAC GTC TCG AAA TCA TCC ATT GCT GCA CAG GA-3’ and 5’-CAC GTC TCA TTA AAA TGA AAT GGG GGA TGG AA-3’
Library 8 vector: 5’-CAC GTC TCA GGC CTT GAC ACA TGG CCT ATG GC-3’ and 5’-CAC GTC TCC CAC AAC TAG AAG GAT TTT CAG CT-3’
Library 9 vector: 5’-CAC GTC TCC CTT CCC AAT GGA ACC TTC CTC CA-3’ and 5’-CAC GTC TCT GTC CAA AAA GTA CCT TGT TTC TA-3’

The PCR was performed using KOD DNA polymerase (EMD Millipore, Billerica, MA) with 1.5 mM MgSO4, 0.2 mM of each dNTP (dATP, dCTP, dGTP, and dTTP), 20 ng pHW2000-PA plasmid, and 0.6 uM of forward and reverse primer. The thermocycler was set as follows: 2 minutes at 95°C, then 20 three-step cycles of 20 seconds at 95°C, 15 seconds at 58°C, and 3.5 minutes at 68°C, and a 2 minutes final extension at 68°C. The PCR product was digested by DpnI (New England Biolabs) to remove the input plasmid.

The insert was then digested by BsaI (New England Biolabs, Ipswich, MA), whereas the vector was digested by BsmBI (New England Biolabs). Ligation was performed for each of the nine libraries with T4 DNA ligase (Life Technologies, Carlsbad, CA) using the corresponding insert and vector. Transformations were carried out with electrocompetent MegaX DH10B T1R cells (Life Technologies). For each of the nine mutant libraries, ∼ 50,000 colonies were scraped and directly processed for plasmid DNA purification (Qiagen Sciences, Germantown, MD). Point mutations for the validation experiment were constructed using the QuikChange XL Mutagenesis kit (Stratagene) according to the manufacturer’s instructions.

### Transfections, infections and titering

∼ 35 million 293T (human embryonic kidney) cells in a 175 cm^2^ flask were used for transfection to rescue each viral mutant library from the plasmid mutant library as described [44–46]. Transfections were performed using Lipofectamine 2000 (Life Technologies) according to the manufacturer’s instructions. Supernatant was replaced with fresh cell growth medium at 24 hours and 48 hours post-transfection. At 72 hours post-transfection, supernatant containing infectious virus was harvested, filtered through a 0.45 um MCE filter, and stored at -80°C. The TCID_50_ was measured on A549 cells (human lung carcinoma cells). For infection, ∼ 10 million A549 cells in a 50 cm^2^ plate were used with an MOI of 0.05. At 2 hours post-infection, infected cells were washed three times with PBS followed by the addition of fresh cell growth medium. Virus was harvested at 24 hrs post-infection.

### Sequencing library preparation

Viral RNA was extracted using QIAamp Viral RNA Mini Kit (Qiagen Sciences) and treated with DNaseI (Life Technologies) to digest any residual plasmid DNA from transfection. The DNA-free RNA was then reverse transcribed to cDNA using Superscript III reverse transcriptase (Life Technologies). The plasmid mutant libraries or cDNA from the viral mutant libraries (transfection or infection) were amplified using the following primers:

Library 1: 5’-CTG ATT CTG GAG GGA AGA TTT TGT GCG A-3’ and 5’-TGC AAA CTG GAG TTA TTA CTG TCC AGG C-3’
Library 2: 5’-AAT AAT CTG GAG AAG AGA TCG CAC AAT A-3’ and 5’-ATA GCC CTG GAG TGA TCC TAG CCC TGC T-3’
Library 3: 5’-AAA GGC CTG GAG CAC TCT CGA TGA AGA A-3’ and 5’-TAG CAT CTG GAG CTT TGG ACA TTT GAG A-3’
Library 4: 5’-ACC GAA CTG GAG CAT TGA GGG CAA GCT T-3’ and 5’-GAA GAT CTG GAG GAT TTA TTC CCT TTT C-3’
Library 5: 5’-GAA GGA CTG GAG TGT TGT TAA ACC ACA C-3’ and 5’-CAC ATG CTG GAG TGA ACT CAT TCT GAA T-3’
Library 6: 5’-ACC AGA CTG GAG GTC GCT TGC AAG TTG G-3’ and 5’-GCC TTC CTG GAG TAG TTC TAC ACT TGC T-3’
Library 7: 5’-GGA TGA CTG GAG ATT AAT TCC AAT GAT A-3’ and 5’-TCA TTT CTG GAG TTG AGG TTC CAT TTG T-3’
Library 8: 5’-GTC AAG CTG GAG GTT CTT GTA TGT GAG G-3’ and 5’-CTA GTT CTG GAG ATG CAT ACA AGC TGT T-3’
Library 9: 5’-ATT GGG CTG GAG TGC AGA ACT TTA TTG G-3’ and 5’-TTT TTG CTG GAG ATG GAT AGC AAA TAG T-3’

The PCR was performed using KOD DNA polymerase (EMD Millipore) with 1.5 mM MgSO4, 0.2 mM of each dNTP (dATP, dCTP, dGTP, and dTTP) and 0.6 uM of forward and reverse primer. The thermocycler was set as follows: 2 minutes at 95°C, then 30 three-step cycles of 20 seconds at 95°C, 15 seconds at 54°C, and 20 seconds at 68°C, and a 1 minute final extension at 68°C. The resulting PCR amplicons were digested with BpmI (New England Biolabs). End repair and 3’ dA-tailing were performed by end repair module and dA-tailing module respectively (New England BioLabs). dA-tailed amplicons were ligated to sequencing adapters using T4 DNA ligase (Life Technologies). Adapters were generated by annealing two oligos: 5’-ACA CTC TTT CCC TAC ACG ACG CTC TTC CGA TCT NNN T-3’ and 5’-/5Phos/NNN AGA TCG GAA GAG CGG TTC AGC AGG AAT GCC GAG-3’. The location of multiplex ID for distinguishing different samples is underlined. The nucleotide sequences for the multiplex ID were the reverse complement in the two oligos (S2 Table). The adapter-ligated products were enriched by a final PCR using primers: 5’-AAT GAT ACG GCG ACC ACC GAG ATC TAC ACT CTT TCC CTA CAC GAC-3’ and 5’-CAA GCA GAA GAC GGC ATA CGA GAT CGG TCT CGG CAT TCC TGC TGA ACC-3’. This final PCR was performed using KOD DNA polymerase (EMD Millipore) with 1.5 mM MgSO4, 0.2 mM of each dNTP (dATP, dCTP, dGTP, and dTTP) and 0.6 uM of forward and reverse primer. The thermocycler was set as follow: 2 minutes at 95°C, then 18 three-step cycles of 20 seconds at 95°C, 15 seconds at 56°C, and 20 seconds at 68°C, and a 1 minute final extension at 68°C. Deep sequencing was performed using two lanes of the Illumina MiSeq with 250 bp paired-end reads. Raw sequencing data have been submitted to the NIH Short Read Archive (SRA) under accession number: BioProject PRJNA254185.

### Sequencing data analysis

Sequencing data were de-multiplexed by the three-nucleotide barcode. A paired-end read was filtered and removed if the corresponding forward and reverse reads did not match. Each mutation was called by comparing individual reads to the WT reference sequence. All analysis was performed by custom python scripts, which are available upon request. For the RF index calculation, only mutants that carried a single mutation were considered. RF index for a given mutation was computed as follows:

For a mutation i in mutant library n of sample t (where t could be input plasmid library transfection or infection):

Occurrence frequency_*i*,*n*,*t*_ = Read count_*i*,*n*,*t*_/Coverage_*n*,*t*_, where Read count_*i*,*n*,*t*_ represented the number of read in mutant library n of sample t that carried mutation i and coverage_*n*_ represented the sequencing coverage of the mutant library n of sample t.

Similarly, Occurrence frequency_*WT*,*n*,*t*_ = Read count_*WT*,*n*,*t*_/Coverage_*n*,*t*_, where Read count_*WT*,*n*_ represented the number of read that has a complete match with the reference sequence in mutant library n of sample t and coverage_*n*_ represented the sequencing coverage of the mutant library n of sample t.

For a mutation i in mutant library n of sample t:

Relative frequency_*i*,*n*,*t*_ = (Occurrence frequency_*i*,*n*,*t*_)/(Occurrence frequency_*WT*,*n*,*t*_).

Subsequently, RF index = (Relative frequency_*i*,*n*,*infection*_)/(Relative frequency_*i*,*n*,*plasmidlibrary*_)

To avoid fitness calculations being obscured by sequencing errors, only the point mutations with an occurrence frequency of ≥ 0.03% in the DNA library were included in the downstream analysis unless otherwise stated. The RF index for individual mutations is shown in S1 Dataset.

### ΔΔG predictions for single amino acid substitutions

PDB: 4M5Q (PA N-terminal endonuclease domain) [37] and PDB: 2ZNL (PA C-terminal domain) [23] were used for ΔΔG prediction of single amino acid substitution. ΔΔG prediction was performed by the ddg_monomer application in Rosetta software [59]. Parameters from row 16 of Table I in Kellogg *et al*. were used [60]. Briefly, a “soft-rep” energy function was used for side chain repacking for all residues, in which the Lennard-Jones repulsive interactions at short atomic separations were damped. After repacking, a restrained quasi-Newton minimization step was performed for both side chain and backbone using a “hard-rep” energy function, in which the repulsive interactions were not damped. All options followed the high resolution protocol flags of the ddg_monomer application. The ΔΔG prediction result is shown in S2 Dataset. Minimal, if any, destabilizing effect is expected if predicted ΔΔG is < 0.

### Relative solvent exposure surface area (SASA) calculation

DSSP (http://www.cmbi.ru.nl/dssp.html) was used to compute the SASA from the PDB structure [87]. SASA was then normalized to the empirical scale reported in [88]. All terminal residues were excluded from this analysis.

### Luciferase reporter assay for influenza polymerase activity

An influenza A virus-inducible luciferase reporter assay was used to measure the virus polymerase activity [63]. 293T cells seeded on 48-well plates were transfected with 100 ng each of PB2, PB1, PA, NP, 50 ng of vLuciferase reporter plasmid and 5 ng of PGK-renilla-luciferase using Lipofectamine 2000 (Life Technologies) according to the manufacturer’s instructions. Luciferase activity measurement was performed at 24 hours post-transfection using Promega Dual-Luciferase Assay Kit according to the manufacturers instructions (Promega, Madison, WI). Relative luciferase activity was calculated by normalizing the firefly-luciferase activities to their internal renilla luciferase controls.

### Protein expression analysis

293T cells seeded on a 12-well plate were transfected with pHW2000-PA plasmid using Lipofectamine 2000 (Life Technologies) according to the manufacturer’s instructions. At 24 hours post-transfection, cells were lysed and heated with SDS loading buffer for five minutes. Lysates were loaded onto a 10% polyacrylamide gel and subjected to immunoblot analysis. Rabbit anti-PA antibody (catalog number: GTX125932, GeneTex, Irvine, CA), mouse anti-Flag antibody (Sigma), mouse anti-actin antibody ACTN05 (C4) (Abcam, Cambridge, MA), sheep horseradish peroxidase-conjugated anti-mouse Immunoglobulin G (GE Healthcare, Pasadena, CA), and donkey horseradish peroxidase-conjugated anti-rabbit Immunoglobulin G (GE Healthcare) were used for protein detection.

### Real-time reverse-transcription PCR (RT-qPCR)

Viral RNA was extracted using QIAamp Viral RNA Mini Kit (Qiagen Sciences) and treated with DNaseI (Life Technologies) to digest any residual plasmid DNA from transfection. The DNA-free RNA was then reverse transcribed to cDNA using Superscript III reverse transcriptase (Life Technologies). The cDNA was subjected to qPCR analysis. qPCR was performed on a DNA Engine OPTICON 2 system (Bio-Rad, Irvine, CA) using SYBR Green (Life Technologies) with primers: 5’-GAC GAT GCA ACG GCT GGT CTG-3’ and 5’-ACC ATT GTT CCA AC TCC TTT-3’.

### Hemagglutination (HA) assay

HA titer was measured by HA assay. A round-bottom 96-well plate was employed for this assay. A 2-fold serial dilution of the virus was performed using PBS. Different dilutions were then inoculated with a final concentration of 0.25% of turkey red blood cell (Lampire Biological Laboratories, Pipersville, PA) for 30 to 60 minutes at room temperature. Those wells with a uniform reddish color were scored as a positive result.

### Sequence entropy calculation

PA protein sequences of type A and B influenza virus and P3 protein sequences of type C influenza virus were retrieved from the Influenza Research Database [89]. A total of 3271 PA protein sequences from type A influenza virus, 562 PA protein sequences from type B influenza virus, and 4 P3 protein sequences from type C influenza virus were obtained using the following parameters: human host, all geographical locations, complete segment only, include pH1N1, remove duplicate sequences. Multiple sequence alignment was performed along with the A/WSN/33 PA sequence using MAFFT (version 7.157b) [90] using the “–nofft” option. Shannon’s entropy for each residue position was then calculated by:

$\text{Entropy}=-\sum_{i=1}^{M}{\text{P}}_{i}{\text{log}}_{2}(P_{i})$ [66], where P_*i*_ is the fraction of residues of amino acid type i, and M is the number of amino acid types (i.e. 20).

### Phylogenetic tree reconstruction

Amino acid sequences were align with MAFFT (version 7.157b) [90] using default parameters. Phylogenetic tree was generated by FastTree (version 2.1.8) [91] from the sequence alignment using default parameters and displayed in FigTree (version 1.3.1) (http://tree.bio.ed.ac.uk/software/figtree/).

### Computing dN/dS

8726 influenza A PA coding sequences (CDS) were retrieved from the Influenza Research Database [89] using the following parameters: human host, all geographical locations, complete segment only, include pH1N1, remove duplicate sequences, length of 2151 bp. Due to the large amount of computational power required to process such a large number of sequences, 3000 sequences were randomly sampled for dN/dS calculation. Multiple sequence alignment was performed along with the A/WSN/33 PA CDS using MAFFT (version 7.157b) [90] using the “–nofft” option. Phylogenetic tree was generated by FastTree (version 2.1.8) [91] from the sequence alignment using default parameters. The sequence alignment and the phylogenetic tree were analyzed by FUBAR [67] using HyPhy [92]. From the FUBAR output, dN/dS for each codon was calculated by beta/alpha, where beta was the posterior mean non-synonymous substitution rate and alpha was the posterior mean synonymous substitution rate.

### Functional residue prediction from FRpred and firestar

WSN PA protein sequence was used as the input for the firestar server (http://firedb.bioinfo.cnio. es/Php/FireStar.php) [69] using default parameters. For the functional site prediction using FRpred [70, 71], two classification schemes were employed, FRcons and FRsubtype, respectively. For FRcons, a random subset of 2000 aligned PA protein sequences from type A influenza virus was used as input due to the limitation of computational cost. For FRsubtype, 1702 PA protein sequences from type A influenza virus, 294 PA protein sequences from type B influenza virus, and 4 P3 protein sequences from type C influenza virus were used as input. Default parameters were used. Each PA residue was assigned a FRcons category and a FRsubtype category, ranging from 1 to 9, with 1 being least likely to be a functional residue, and 9 being most likely to be a functional residue. In this study, residues that were assigned a category of ≥ 8 were identified as a hit. A total of 72 residues were identified under each of the FRcons classification and the FRsubtype classification.

## Supporting Information

S1 Fig Experimental design. Replicate 1 and replicate 2 of the DNA library were prepared independently as a technical replicate for sequencing. The rescue of the viral mutant library was performed twice as a biological replicate. Infection in A549 cells was performed for 24 hours using the viral mutant library from replicate 1. A biological replicate of infection was performed using the same viral mutant library.
(EPS)

S2 Fig A schematic representation of the sequencing read. Both forward and reverse read of the 250 bp paired-end Illumina MiSeq sequencing cover the 3 bp multiplex sequencing sample identifier (MID) on the 5’ adapter, the 5’ adapter TA ligation site, the 240 bp region of interest, the 3’ adapter TA ligation site, and the 3 bp multiplex sequencing sample identifier (MID) on the 3’ adapter.
(EPS)

S3 Fig Sequencing coverage. Sequencing coverages (number of paired-end reads) of individual mutant libraries from different samples are shown.
(EPS)

S4 Fig Composition of the “small libraries”. Mutation rates in each of the nine plasmid mutant libraries are shown.
(EPS)

S5 Fig Sequencing error rates. Frequency of each individual point mutation is shown. Orange represents the point mutation in the plasmid mutant library. Blue represents the point mutation in the WT plasmid. Point mutations were sorted by frequency in the plasmid mutant library. The frequency distributions are shown as boxplots. The red dashed line refers to the 0.03% cutoff in the mutant library.
(EPS)

S6 Fig Individually constructed substitutions on PA C-terminal domain. The locations of substitution with an RF index < 0.15 and a predicted ΔΔG < 0 are colored in orange or red, respectively. Mutations that were individually reconstructed and analyzed in this study are labeled and colored in red. Residues that were not covered in our profiling data are colored in grey. PB1 is colored in green.
(EPS)

S7 Fig Functional characterization of E430G and R512W. (A) The copy number of influenza NP in supernatant was quantified by qPCR during an eight-plasmid viral rescue experiment. (B) The intracellular copy number of influenza NP was quantified by qPCR during an eight-plasmid viral rescue experiment. (C) HA titer in the viral rescue experiment was measured at 72 hours post-transfection using turkey red blood cell.
(EPS)

S8 Fig PB1 and PB2 interaction surface on PA. (A) Interaction between the subunits PA and PB1. For PA, mutations that were individually reconstructed and analyzed in this study are labeled and colored in red. Residues that were not covered in our profiling data are colored in grey. PB1 is shown in green stick form. RNA is removed for visualization purpose. PDB: 4WSB [64]. (B) Interaction between the subunits PA and PB2. RNA is highlighted in yellow. For PA, mutations that were individually reconstructed and analyzed in this study are labeled and colored in red. Residues that were not covered in our profiling data are colored in grey. PB2 is shown in teal stick form. RNA is removed for visualization purpose. PDB: 4WSB [64].
(EPS)

S9 Fig Structural analysis of PA R512. Interaction between PA R512 and RNA is shown. RNA is colored in yellow. Hydrogen bonds are represented by black dotted lines. PDB: 4WSB [64].
(EPS)

S10 Fig Structural analysis of PA D426, E427, and E430. Location of PA D426, E427 and E430 are shown on the structure of the influenza polymerase heterotrimeric complex (PDB: 4WSB) [64]. Interaction of D426 and E427 with PB1 is shown. PB1 is colored in green. PB2 is colored in cyan. PA is colored in grey. Hydrogen bonds are represented by black dotted lines. PDB: 4WSB [64].
(EPS)

S11 Fig Phylogenetic tree of different types of influenza viruses. Phylogenetic tree reconstruction of A/WSN/33 PA protein sequence, 50 representative PA protein sequences of type A influenza virus, 50 representative PA protein sequences of type B influenza virus, and 4 representative P3 protein sequences of type C influenza virus. The branch that represents A/WSN/33 is colored in red. Branches that represent type A influenza virus are shaded in green. Branches that represent type B influenza virus are shaded in blue. Branches that represent type C influenza virus are shaded in orange. Scale bar indicates the number of substitutions per site.
(EPS)

S12 Fig Comparison of sequence entropy between functional residues, structural residues and “other” residues. (A) Average entropy for functional residues, structural residues and “other” residues are shown. (B) The rank of different categories of residues in the order of low to high entropy (from left to right). The p-value for the difference between different categories of residues are shown. Wilcoxon rank-sum test was performed to compute the p-value.
(EPS)

S13 Fig Comparison between dN/dS and RF index. (A) The relationships between log_10_ RF index for individual amino acid subsitutions and the dN/dS of their corresponding residues are shown. The Spearman’s rank correlation between dN/dS and RF index is 0.38 (P < 2.2e^−16^). (B) Average dN/dS for functional residues, structural residues and “other” residues are shown. The rank of different categories of residues in the order of low to high dN/dS (from left to right). The p-value for the difference between different categories of residues are shown. Wilcoxon rank-sum test was performed to compute the p-value.
(EPS)

S14 Fig PA-RNA interaction in the influenza A virus. The interaction between PA of the influenza A virus and the first five nucleotide in the RNA molecule is plotted in a two-dimensional diagram using LigPlot^+^[95]. Red: oxygen atom; Blue: nitrogen atom; Grey: carbon atom. RNA is colored in cyan. Hydrogen bonds between PA and the RNA molecule are indicated by dotted lines. Atoms that are involved in hydrophobic contact between PA and the RNA molecule are surrounded with cyan spikes. PDB: 4WSB [64]. Numbering of residue position is based on A/WSN/33. Conversion of residue position numbering is described in S3 Table.
(EPS)

S15 Fig PA-RNA interaction in the influenza B virus. The interaction between PA of the influenza B virus and the first five nucleotide in the RNA molecule is plotted in a two-dimensional diagram using LigPlot^+^[95]. Red: oxygen atom; Blue: nitrogen atom; Grey: carbon atom. RNA is colored in cyan. Hydrogen bonds between PA and the RNA molecule are indicated by dotted lines. Atoms that are involved in hydrophobic contact between PA and the RNA molecule are surrounded with cyan spikes. PDB: 4WSA [72]. Numbering of residue position is based on A/WSN/33. Conversion of residue position numbering is described in S3 Table.
(EPS)

S1 Table Known functional residues of PA. A list of 28 functional residues that have been described in the literature are shown. This list serves as a benchmark for comparing different approaches for identifying functional residues. “Substitutions sampled” indicates those amino acid substitutions with the fitness effect being profiled in this study.
(PDF)

S2 Table Sequence of the multiplex ID. The nucleotide sequences of multiplex ID for identifying different samples in the deep sequencing experiment are listed. These nucleotide sequences represent the first three nucleotides of both forward and reverse sequencing reads.
(PDF)

S3 Table Conversion of residue position numbering. The residue position numbering in the PDB file (4WSB and 4WSA) is slightly different from WSN PA sequence. The locations of those residues that are being mentioned in this study are listed.
(PDF)

S1 Dataset Fitness profiling result. The RF index of each point mutation being profiled in this study is listed.
(XLS)

S2 Dataset ΔΔG prediction result. The predicted ΔΔG for each substitution is listed.
(XLS)
