# Correction: Functional Constraint Profiling of a Viral Protein Reveals Discordance of Evolutionary Conservation and Functionality

**DOI:** 10.1371/journal.pgen.1006709

**Published:** 2017-04-25

**Authors:** Nicholas C. Wu, C. Anders Olson, Yushen Du, Shuai Le, Kevin Tran, Roland Remenyi, Danyang Gong, Laith Q. Al-Mawsawi, Hangfei Qi, Ting-Ting Wu, Ren Sun

There is a labelling error in Panel C of Fig 2. The labels ‘PA N-terminal Domain’ and ‘PA C-terminal Domain’ are swapped. The left label should be ‘PA N-terminal Domain’, and the right label should be ‘PA C-terminal Domain’. The authors have provided the correct version of Fig 2 which can be viewed below.

**Fig 2:**
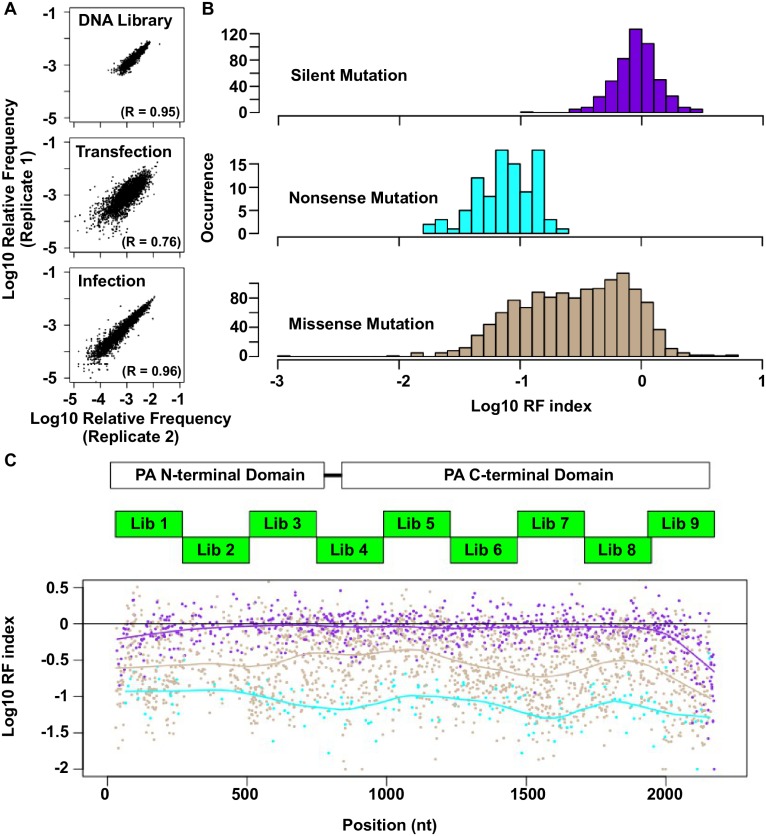
Fitness profiling of PA influenza virus polymerase subunit. (A) Correlations of log_10_ relative frequency of individual point mutations between replicates are shown. Relative frequency_*mutation i*_ = (Occurrence frequency_*mutation i*_)/(Occurrence frequency_*WT*_) (B) Log_10_ RF indices for silent mutations, nonsense mutations, and missense mutations are shown as histograms. Point mutations located at the 5 terminal 400 bp and 3 terminal 400 bp regions are not included in this analysis to avoid complication by the vRNA packaging signal [93, 94]. (C) The locations of the PA C-terminal domain and the PA N-terminal domain are shown as white boxes. The locations of the mutated regions in each mutant library are shown as green boxes. Log_10_ RF indices for individual point mutations are plotted across the PA gene. Each point mutation is colored coded as in panel B. Purple: silent mutations; Cyan: nonsense mutations; Brown: missense mutations. A smooth curve was fitted by loess and plotted for each point mutation type.
