# Supplementary material for: Functional Constraint Profiling of a Viral Protein Reveals Discordance of Evolutionary Conservation and Functionality
Source: PLoS Genet. 2015 Jul 1;11(7):e1005310. doi: 10.1371/journal.pgen.1005310 (PMC4489113; doi:10.1371/journal.pgen.1005310)
Supplement: S1 Table — A list of 28 functional residues that have been described in the literature are shown. This list serves as a benchmark for comparing different approaches for identifying functional residues. “Substitutions sampled” indicates those amino acid substitutions with the fitness effect being profiled in this study. (PDF) [file pgen.1005310.s016.pdf]

S1 Table

| | | | This study | | firestar | frcons | frsubtype |
|---|---|---|---|---|---|---|---|
| Residue | Function | Reference | Substitutions sampled | Identified | Identified | Category | Category |
| H41 | endonuclease active site | [20, 21] | L, R | | Yes | 8 | 1 |
| E80 | endonuclease active site | [20, 21] | G, K, V | Yes | Yes | 3 | 1 |
| D108 | endonuclease active site | [20, 21] | - | | Yes | 4 | 1 |
| E119 | endonuclease active site | [20, 21] | E, G, V | Yes | Yes | 5 | 1 |
| K134 | endonuclease active site | [20, 21] | M | Yes | Yes | 2 | 1 |
| D164 | Polymerase activity | [61] | G, N | | | 3 | 1 |
| E165 | Polymerase activity | [61] | D, G, V | | | 3 | 1 |
| E166 | Polymerase activity | [61] | D, G | Yes | | 3 | 1 |
| R170 | Polymerase activity | [61] | G, K, M, W | Yes | | 4 | 1 |
| K172 | Polymerase activity | [61] | E, I, K, N, R | | | 4 | 9 |
| T173 | Polymerase activity | [61] | A, I | Yes | | 9 | 5 |
| F176 | Polymerase activity | [61] | L, S, Y | | | 4 | 8 |
| K378 | Viral growth | [33] | E, R, M | | | 7 | 1 |
| D478 | Viral growth | [33] | G, N, V | | | 1 | 2 |
| G502 | Polymerase activity | [28] | C, D, S | | | 9 | 1 |
| H510 | endonuclease activity | [28] | R, L, Y | Yes | | 9 | 1 |
| E524 | Polymerase activity | [28] | G, K, V | Yes | | 5 | 1 |
| K539 | Polymerase activity | [28] | E, R, M | Yes | | 4 | 1 |
| R566 | binding to svRNA | [62] | G, W | Yes | | 3 | 1 |
| S624 | Protease activity | [34] | P, F | | | 3 | 7 |
| V636 | PB1-binding | [23] | A, D, I | | | 6 | 2 |
| R638 | Polymerase activity | [28, 29] | G, R, S | | | 2 | 1 |
| L640 | PB1-binding | [23] | I | | | 4 | 4 |
| E656 | Polymerase activity, viral growth | [33] | D, G | | | 4 | 1 |
| L666 | PB1-binding | [22, 23] | F, H, P | | | 1 | 1 |
| Q670 | PB1-binding | [22] | H, L, Q, R | Yes | | 3 | 2 |
| W706 | PB1-binding | [22, 23] | R | | | 3 | 2 |
| F710 | PB1-binding | [22] | I, L, S, Y | Yes | | 3 | 3 |
