# Supplementary material for: Functional Constraint Profiling of a Viral Protein Reveals Discordance of Evolutionary Conservation and Functionality
Source: PLoS Genet. 2015 Jul 1;11(7):e1005310. doi: 10.1371/journal.pgen.1005310 (PMC4489113; doi:10.1371/journal.pgen.1005310)
Supplement: S2 Table — The nucleotide sequences of multiplex ID for identifying different samples in the deep sequencing experiment are listed. These nucleotide sequences represent the first three nucleotides of both forward and reverse sequencing reads. (PDF) [file pgen.1005310.s017.pdf]

S2 Table

Sample	Multiplex ID
WT Control	TCG
DNA library (Replicate 1)	TAA
DNA library (Replicate 2)	ACT
Transfection (Replicate 1)	AGA
Transfection (Replicate 2)	ATG
Infection (Replicate 1)	GAG
Infection (Replicate 2)	CTT
