# Supplementary material for: Functional Constraint Profiling of a Viral Protein Reveals Discordance of Evolutionary Conservation and Functionality
Source: PLoS Genet. 2015 Jul 1;11(7):e1005310. doi: 10.1371/journal.pgen.1005310 (PMC4489113; doi:10.1371/journal.pgen.1005310)
Supplement: S3 Table — The residue position numbering in the PDB file (4WSB and 4WSA) is slightly different from WSN PA sequence. The locations of those residues that are being mentioned in this study are listed. (PDF) [file pgen.1005310.s018.pdf]

S3 Table

Residue	A/WSN/33	4WSB (type A)	4WSA (type B)
281	K281	**K281**	A281
326	H326	**H326**	S328
328	K328	**K328**	**K330**
368	W368	**W363**	**W364**
369	A369	**G364**	**A365**
370	L370	L365	**T366**
371	G371	**G366**	**G367**
374	M374	**Q369**	**L370**
396	D396	E391	**K392**
398	P398	**P393**	**P394**
517	V517	**V512**	**V513**
519	N519	**N514**	**T515**
539	K539	**K534**	K535
559	R559	K554	K551
566	R566	**R561**	**R558**
567	T567	**T562**	**V559**
568	N568	**N563**	**N560**
569	G569	**G564**	**G561**
574	K574	**K569**	**Q566**
696	N696	N691	**N692**

*Residues involving in RNA binding as determined by LigPlot^{+} are in bold
